# GETTING TO KNOW YOU: USING VIRTUAL REALITY IN VOLUNTEER RESPITE PROGRAM FOR PERSONS WITH MEMORY LOSS

**DOI:** 10.1093/geroni/igad104.3372

**Published:** 2023-12-21

**Authors:** Louanne Bakk, Lillian Agyemang, Mary Milnamow, Jason Dauenhauer, Katy Allen

**Affiliations:** University at Buffalo, Buffalo, New York, United States; University at Buffalo School of Social Work, Buffalo, New York, United States; University at Buffalo, Buffalo, New York, United States; SUNY Brockport, Brockport, New York, United States; Lifespan of Greater Rochester, Rochester, New York, United States

## Abstract

Virtual reality (VR) can provide an immersive experience that simulates real or imagined environments and provides an innovative solution to promote engagement and socialization. Interventions to date have not explored the use of trained volunteers to use VR to promote socialization and provide active experiences with community-dwelling persons with memory loss. This presentation will provide an overview of a volunteer-led drop-in respite caregiving program. The program uses Rendever’s VR platform, which includes a series of pre-programmed and on-demand immersive experiences delivered to persons with memory loss through a VR headset via a volunteered-controlled tablet. Volunteers conduct tailored individual and group VR sessions as a scheduled activity with persons with memory loss. This study aimed to understand the experiences of volunteers administering VR programming within the drop-in respite center. A total of eight volunteers participated in a focus group. A semi-structured interview guide explored volunteers’ motivation for becoming trained to use VR, observations of using VR with persons with memory loss, how they are implementing VR, challenges they experienced using VR, and ideas for improving/expanding implementation. Volunteers reported increased socialization when using VR with persons with memory loss, a desire for continued and ongoing VR training, and suggested larger group VR sessions delivering the same content to foster greater interaction among respite participants. Results suggest that VR programming can be an effective format for increasing socialization and involvement among persons living with memory loss.

